# Patent Medicine

**Published:** 1854-02

**Authors:** 


### PATENT MEDICINE.

The following certificate to the efficacy of Patent Pills is taken from the Phil. Mercury.

I, John Lubberlie, was supposed to be in the last stage of consumption in the year '48, suffering at the same time under a severe attack of rheumatism, liver-complaint, gravel, dropsy and cholera morbus. Simultaneously, also, I took yellow fever and small-pox. The latter assuming the chronic form of scrofula, completely destroyed my lungs, liver, spinal marrow, nervous system, and the entire contents of my cranium. I got so low, that I did not know my brother-in-law, when he came to borrow some money. For three months I swallowed nothing but twenty packages of Kunklehausen's pills which effected an immediate cure in two weeks. Sworn and subscribed to, &c.

P. S.—My late uncle, Bacchus Pottinger, was afflicted so long with the gout, (contracted by living too much on bear's meat and alligator's eggs,) that his life became a burden to him. He took only four boxes of said pills, and life was a burden to him no longer.

